# ddRAD sequencing-based identification of inter-genepool SNPs and association analysis in *Brassica juncea*

**DOI:** 10.1186/s12870-019-2188-x

**Published:** 2019-12-30

**Authors:** Jebi Sudan, Ravinder Singh, Susheel Sharma, Romesh K. Salgotra, Varun Sharma, Gurvinder Singh, Indu Sharma, Swarkar Sharma, Surinder K. Gupta, Sajad Majeed Zargar

**Affiliations:** 1 School of Biotechnology, Sher-e-Kashmir University of Agricultural Sciences and Technology of Jammu, Jammu, J&K India; 2 JECRC University- Jaipur, Jaipur, Rajasthan India; 3 Human Genetics Research Group, School of Biotechnology, Shri Mata Vaishno Devi University, Katra, J&K India; 4 Division of Plant Breeding and Genetics, Sher-e-Kashmir University of Agricultural Sciences and Technology of Kashmir, Jaipur, J&K India; 5 Division of Plant Biotechnology, Sher-e-Kashmir University of Agricultural Sciences and Technology of Kashmir, Jammu, J&K India

**Keywords:** Single nucleotide polymorphisms (SNPs), Double digest-*R*estriction *A*ssociated *D*NA (dd-RAD), MassARRAY, Diversity analysis, Association mapping

## Abstract

**Background:**

Narrow genetic base, complex allo-tetraploid genome and presence of repetitive elements have led the discovery of single nucleotide polymorphisms (SNPs) in *Brassica juncea* (AABB; 2n = 4x = 36) at a slower pace. Double digest RAD (ddRAD) - a genome complexity reduction technique followed by NGS was used to generate a total of 23 million paired-end reads from three genotypes each of Indian (*Pusa Tarak*, *RSPR-01* and *Urvashi*) and Exotic (*Donskaja IV*, *Zem 1* and *EC287711*) genepools.

**Results:**

Sequence data analysis led to the identification of 10,399 SNPs in six genotypes at a read depth of 10x coverage among the genotypes of two genepools. A total of 44 hyper-variable regions (nucleotide variation hotspots) were also found in the genome, of which 93% were found to be a part of coding genes/regions. The functionality of the identified SNPs was estimated by genotyping a subset of SNPs on MassARRAY® platform among a diverse set of *B. juncea* genotypes. SNP genotyping-based genetic diversity and population studies placed the genotypes into two distinct clusters based mostly on the place of origin. The genotypes were also characterized for six morphological traits, analysis of which revealed a significant difference in the mean values between Indian and Exotic genepools for six traits. The association analysis for six traits identified a total of 45 significant marker-trait associations on 11 chromosomes of A- and B- group of progenitor genomes.

**Conclusions:**

Despite narrow diversity, the ddRAD sequencing was able to identify large number of nucleotide polymorphisms between the two genepools. Association analysis led to the identification of common SNPs/genomic regions associated between flowering and maturity traits, thereby underscoring the possible role of common chromosomal regions-harboring genes controlling flowering and maturity in *Brassica juncea*.

## Background

*Brassica juncea* commonly known as Indian mustard is an important oilseed crop in Indian subcontinent, northern China and eastern European countries. It is widely and extensively grown for seeds which yield an essential oil and condiment; however its young leaves are also used as vegetables or mixed with other salad greens. *Brassica juncea* has two diverse genepools: the Indian and the east European genepool (exotic) [1]. The east European genepool shows more diversity at the molecular level and has more yield potential while the Indian genepool has narrow genetic diversity with low yield potential [2, 3]. In spite of the two morphological diverse pool, the crop experienced narrow genetic base that might be due to complex allotetraploid genome and domestication [4]. This narrow genetic base has hindered the process of germplasm enhancement as it reduces the chances of finding the diverse alleles of important agronomic traits for their introgression into elite germplasm [5].

The genetic enhancement can be achieved by the transfer of alleles between exotic (European) and Indian genepools using either traditional plant breeding approaches or marker-assisted selection (MAS). While MAS require the identification and use of closely and tightly linked molecular markers with the trait of interest, association mapping does not need prior molecular mapping information and serves as an important tool to identify marker-trait associations on the basis of linkage disequilibrium (LD) only. Association analysis infers significant marker-trait associations by accounting for co-segregated (or co-transmission) alleles at different locations in a genome across a diverse set of mapping population [6], allows fine mapping of traits when used with a dense set of molecular markers. In oilseed *Brassica spp.* (*B. juncea* and *B. napus*) different types of molecular markers were employed with a combination of various models (GLM, Q, PCA and K) to figure out close relationship between various traits and markers. In most of the association mapping studies, the SSR markers were used for population studies due to their usefulness in population genetics inferences and these being highly informative when compared to biallelic markers [7]. However, the high heritability of SNPs also makes them an excellent indicator of genetic diversity and phylogeny in crop species with ancient genome duplications, such as in *B. juncea*. Various SSR-based genome-wide association mapping studies were conducted in *B. juncea* [8, 9] and *B. napus* [10–12] for various agronomically important traits. Moreover, Single Nucleotide Polymorphisms (SNPs) are also preferred for fine mapping studies, as reported in *B. napus* [13–17]. However no such study has been reported in *B. juncea*, mainly due to non-availability of SNP markers.

The discovery of SNPs in *B. juncea* has proceeded with a slower pace mainly due to its narrow genetic base, complex allotetraploid genome and highly repetitive regions [18, 19]. The presence of two sub-genomes (A and B) makes SNP discovery and genotyping more difficult and troublesome due to the presence of both homologous and homoeologous DNA sequences. The process of SNP discovery is further complicated with duplications and triplications of A and B genomes due to polyploidization events [20]. To reduce the complexity of genomes, various genome reduction methods are available that uses a set of restriction enzymes and a particular selection process to sequence only the selected set of restriction fragments from multiple genotypes so as to do both the SNP discovery and genotyping at the same time. Advances in the bioinformatics software also support the rapid identification of true SNPs in the individuals.

In this study, a modified ddRADseq approach was followed to partially sequence genomes of six genotypes (three each from Indian and European genepool) of *B. juncea* for SNPs identification and genotyping. A bioinformatics pipeline was developed using tools available within CLC Genomics workbench for the detection of SNPs (Additional file 1: Figure S1). These SNPs were then used to assess the levels of molecular diversity and population structure among diverse set, and association mapping to identify significant marker-trait associations for six morphological traits.

## Results

### ddRAD-library preparation and sequencing

The microfluidics-based electrophoresis analyses of pooled library revealed that majority of fragments were represented in the range of 300 to 400 bases following size selection during library preparation (Additional file 1: Figure S2). The sequence-based barcoding followed by pooling and sequencing of six genotypes on Illumina HiSeq 2000 platform generated a total of about 23 million paired-end reads with an average of 3.83 million reads per genotypes. The mean read quality (Phred score) of six samples was 35.02 and about 89% reads had a Q score > 30, indicating that most of the raw data were of good quality. The mean quality score of read 1 (R1) was slightly better than mean quality score of read 2 (R2) (Additional file 1: Table-S3). A slightly better quality of read 1 than 2 was attributed to the fact that the clones within each cluster in a flow cell had least damage due to repeated flushing of flow cell. This difference in quality scores between two reads is also attributed to phasing errors [21].

### Sequence analysis and SNP identification

After trimming for low quality sequences, the processed reads were assembled into contigs followed by their alignment to the reference genome of *B. juncea* (GenBank: LFQT00000000) using default parameters and about 92–94% reads were mapped to the reference genome. The mapping percentages for individual samples ranged from 92.56 (Zem 1) to 94.35 (Donskaja IV). Out of all the mapped reads, more than 80% reads mapped uniquely to a single locus (Table 1).

**Table 1:** Summary of SNPs obtained in different genotypes

Samples	Total Reads	Total of reads after pre-processing	No. of reads mapped	Percent reads mapped	Percent uniquely mapped reads	SNPs at 10x coverage
Zem 1	2,310,662	2,291,416	2,156,974	94.13	83.60	1251
Donskaja IV	2,361,678	2,350,360	2,201,589	93.67	83.72	1273
EC- 287711	3,462,876	3,437,132	3,231,248	94.03	84.19	1860
Pusa Tarak	7,521,368	7,438,610	6,885,178	92.56	83.25	2127
Urvashi	3,731,080	3,714,434	3,504,568	94.35	81.37	1898
RSPR-01	3,621,796	3,597,428	3,375,826	93.84	82.50	1990

The alignments of contig sequences to the reference genome were used for the identification of SNPs using Probabilistic variant detection method. After filtering for homoeologs, a total of 10,399 single nucleotide variants with a depth of at least 10 reads were found to be distributed among six genotypes.

### SNPs in hyper-variable regions and protein prediction

The stringent condition followed during size selection and SNPs identification has led to the retrieval of less number of SNP markers in the genotypes. Although the SNPs were distributed on all the chromosomes there were regions in various chromosomes with high frequency of SNPs as compared to the other regions referred to as hyper-variable regions or SNP hotspots. In all, a total of 44 hypervariable regions or hotspots of SNPs were found on all chromosomes except A08 and B03. Total number of hypervariable regions on these chromosomes ranged from one to five and total number of SNPs in these regions ranged from four to eleven. A BlastX analysis of the hyper-variable sequences identified that nearly 93.2% of these hypervariable regions found to be part of coding sequences (Table 2).

**Table 2:** Hypervariable regions on different chromosome of *Brassica juncea*

Chromosome	Hyper-variable region	No. of SNPs	Frequency of SNPs (per bp)	Predicted Protein	Protein Accession ID	BlastX e-value
A01	12,089,878 to 12,089,908	4	7.50	SMAX1-LIKE 2 [*Brassica rapa]*	XP_009127888.1	1.00E-148
	13,064,396 to13064410	6	2.33	Heptahelical transmembrane protein 2 [*Brassica napus*]	XP_013730768.1	2.00E-040
	23,525,876 to 23,525,886	4	2.50	Farnesyl transferase/ geranylgeranyl transferase type-1 subunit alpha [*Brassica rapa*]	XP_009147222.1	2.00E-029
	37,133,315 to 37,133,336	5	4.20	Uncharacterized protein LOC106335203 [*Brassica oleracea*]	XP_013629110.1	1.00E-042
	38,650,911 to 38,650,931	4	5.00	Calcium-binding EF-hand family protein (*Arabidopsis thaliana*)	NP_001332476.1	6.00E-056
A02	2,398,121 to 2398162	6	6.83	No significant hit		
	14,416,494 to 14,416,505	5	2.20	Uncharacterized protein	RQM01316.1	3.00E-030
A03	25,641,944 to 25,641,977	7	4.71	Hypothetical protein DY000_00001393 [*Brassica cretica]*	RQL75529.1	1.00E-009
	42,576,172 to 43,014,367	9	21.66	Uncharacterized protein LOC106363406 [*Brassica napus]*	XP_013658605.1	3.00E-079
A04	8,377,252 to 8,377,288	10	3.60	Uncharacterized protein LOC106360747 [*Brassica napus]*	XP_013655857.1	3.00E-113
	14,731,032 to 14,908,156	4	31.00	Transcription factor MYC2 [*Brassica rapa*)	XP_009151447.1	8.00E-166
A05	1,588,930 to 1,588,970	5	8.00	Polygalacturonase-like [*Brassica oleracea*]	XP_013633855.1	4.00E-084
	19,985,702 to 19,985,780	5	15.60	BnaC08g47040D [*Brassica napus]*	CDY43697.1	8.00E-054
	21,938,359 to 21,938,393	6	5.66	Probable LRR receptor-like serine/threonine-protein kinase At4g36180 [*Brassica napus]*	XP_013716480.1	5.00E-166
	27,265,183 to 27,265,231	5	9.60	Hypothetical protein BRARA_K01418 [*Brassica rapa*]	RIA04352.1	1.00E-019
	35,529,249 to 35,529,274	5	5.00	Cis-phytoenedesaturase, chloroplastic/chromoplastic [*Brassica napus*]	XP_013750375.2	9.00E-065
A06	11,405,649 to 11,405,684	5	7.00	BnaA06g16240D [*Brassica napus*]	CDY08102.1	4.00E-077
	31,624,120 to 31,624,155	6	5.83	Hypothetical protein RQL85806.1	RQL85806.1	2.00E-097
	36,377,825 to 36,377,860	5	7.00	Uncharacterized protein LOC106401547 [*Brassica napus]*	XP_022543736.1	5.00E-084
A07	13,947,963 to 13,948,008	5	9.00	Unnamed protein product (*Brassica rapa*)	VDC98355.1	2.00E-072
	16,375,261 to 16,375,275	5	2.80	Uncharacterized protein LOC103829921 [*Brassica rapa]*	XP_009103854.1	6.00E-034
B01	24,425,289 to 24,425,343	10	5.40	Uncharacterized abhydrolase domain-containing protein DDB_G0269086-like [*Brassica rapa*]		4.00E-090
	33,418,235 to 33,418,261	5	5.20	Unnamed protein product [*Brassica rapa*]	VDC90843.1	2.00E-094
	51,944,663 to 51944714	6	8.50	Uncharacterized protein LOC106308810 [*Brassica oleracea*]	XP_013601383.1	4.00E-098
	53,225,827 to 53,225,840	5	2.60	Homocysteine S-methyltransferase 2 [*Brassica napus]*	XP_013712093.1	1.00E-040
	53,650,933 to 53,650,972	5	7.80	Hypothetical protein DY000_00003913 [*Brassica cretica]*	RQL77992.1	4.00E-076
B02	6,040,643 to 6,040,712	11	6.27	Unnamed protein product [*Brassica rapa*]	VDD17261.1	4.00E-004
	14,431,647 to 14,431,685	5	7.60	No significant hit		
B04	18,482,342 to 18,482,397	4	13.75	Uncharacterized protein At3g60930, chloroplastic-like [*Brassica napus*]	XP_022567433.1	7.00E-009
	29,636,717 to 29,636,764	8	5.87	BnaA02g16800D [*Brassica napus]*	CDY49126.1	8.00E-035
	46,447,876 to 46,447,889	6	2.16	hypothetical protein DY000_00030648 [Brassica cretica]	RQM04119.1	4.00E-034
	52,254,291 to 52,254,327	5	7.20	ras-related protein RABD2c isoform X1 [*Brassica rapa*]	XP_009130704.1	1.00E-154
	53,387,066 to 53,387,117	5	10.20	uncharacterized mitochondrial protein AtMg00810-like [*Brassica oleracea var. oleracea*]	XP_013601341.1	2.00E-138
B05	12,937,005 to 12,937,060	5	10.00	hypothetical protein MANES_08G079400 [*Manihot esculenta*]	OAY43565.1	3.00E-014
	68,021,640 to 68,021,664	7	3.42	caffeic acid 3-O-methyltransferase-like [*Raphanus sativus*]	XP_018461242.1	4.00E-058
	80,717,492 to 80,717,516	7	3.43	glutathione S-transferase T3-like [*Brassica oleracea* var. oleracea]	XP_013639324.1	3.00E-107
B06	1,593,067 to 1,593,109	11	3.82	BnaC04g03580D [*Brassica napus*]	CDY17861.1	8.00E-011
	2,770,283 to 2,770,305	5	4.40	uncharacterized acetyltransferase At3g50280 [*Raphanus sativus*]	XP_018434505.1	0
B07	23,121,758 to 23121773	5	3.00	uncharacterized protein LOC108830363 [*Raphanus sativus*]	XP_018459472.1	1.00E-150
B08	15,772,508 to 15,772,583	10	7.50	probable LRR receptor-like serine/threonine-protein kinase At4g36180 isoform X1 [*Raphanus sativus]*	XP_018460684.1	2.00E-074
B09	29,343,310 to 29,343,368	8	7.25	No significant hit	NA	NA
	32,306,181 to 32,306,211	6	5.00	U-box domain-containing protein 9-like [*Brassica napus*]	XP_013684649.1	4.00E-055
B10	63,679,073 to 63,679,088	6	2.50	uncharacterized protein LOC106424516 [*Brassica napus*]	XP_013720737.1	1.00E-107
	69,805,021 to 69,805,080	7	8.42	uncharacterized protein LOC106361641 [*Brassica napus*]	XP_022549137.1	0

### Morphological analysis of diverse genepools

**Fig. 1:**
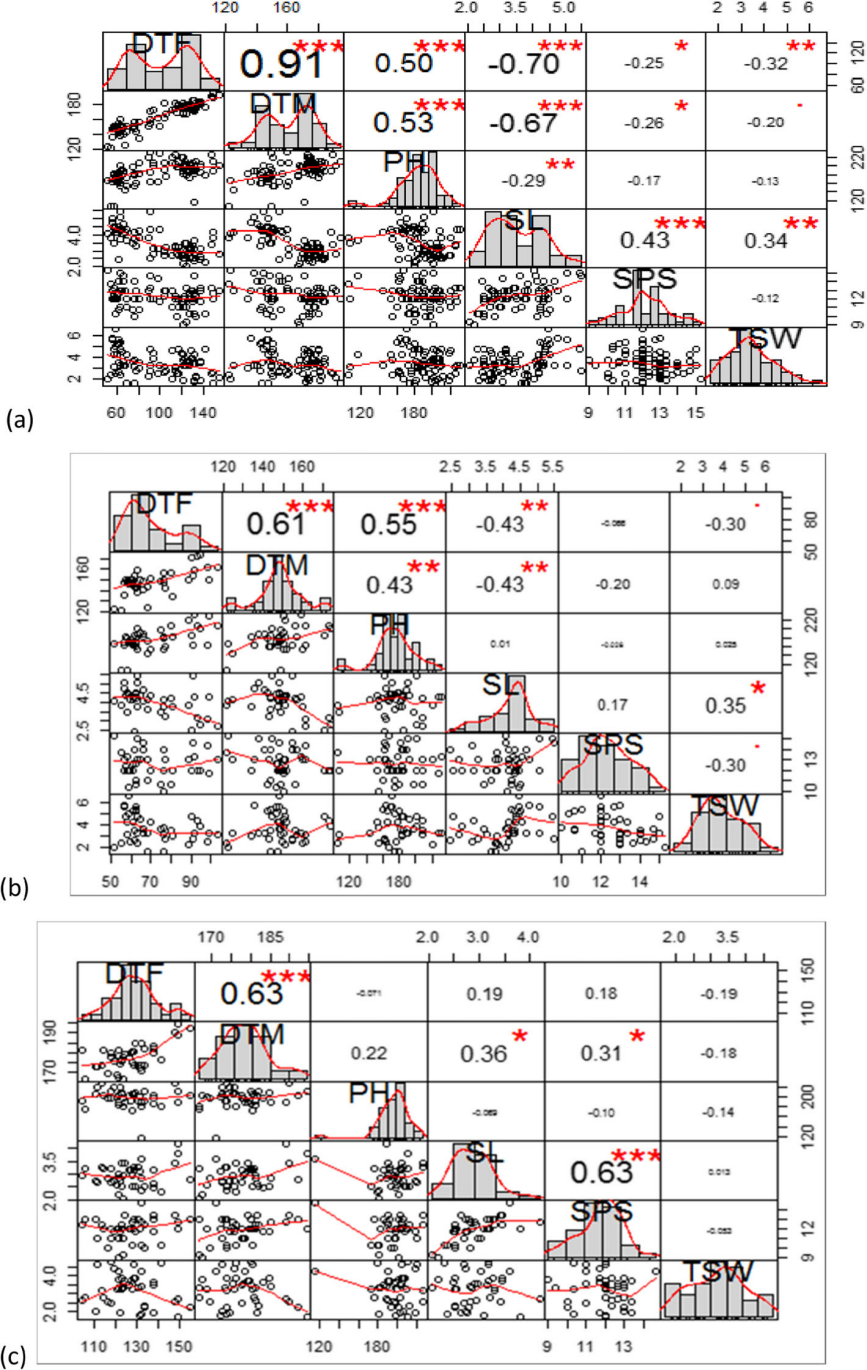
Correlation chart for six morphological traits: (**a**) using 80 genotypes of *Brassica juncea* of Indian and European genepools indicating bi-modal distribution for DTF and DTM (**b**) of genotypes of Indian genepool only, and (**c**) of genotypes of European genepool only

The diverse core set of *B. juncea* consisting of 80 genotypes was characterized for various growth and yield traits under two locations in 2015–16 and 2016–17. The data collected over two locations was used to calculate mean values for individual genotypes for days to flowering (DTF), days to maturity (DTM), plant height (PH), siliqua length (SL), seeds per siliqua (SPS) and thousand seed weight (TSW). An analysis of chart correlation for various traits indicated that except DTF and DTM, all the traits were normally distributed in the diverse core set (Fig. 1). In view of the bi-modal distribution for DTF and DTM, the average values for these two traits were used to classify the diverse core set into two genepools namely European (Exotic) and Indian genepools. The individual chart correlation for two genepools indicated normal distribution for all the traits including DTF and DTM**.**

Among two genepools, the traits had divergent correlations with other traits. In Indian genepool, DTF had high correlation with all traits except SPS; while DTF in European genes had significant correlation with DTM only. The DTM in Indian genepool was significantly correlated with PH (0.43) and SL (− 0.43); however the same trait did not show any significant correlation with PH and a positive significant correlation with SL (0.36) and SPS (0.31). PH was significantly correlated with DTF and DTM in Indian genepool but not in European. SL was negatively correlated with both DTF and interestingly with DTM; and positively with TSW in Indian genepool. However, SL had significant but opposite correlation with DTM in European genepool. SPS was not correlated with any of the yield traits in Indian genepool, but was significantly correlated with SL and DTM. In both the genepools, TSW was not significantly correlated with any of the traits but SL in European. The t-test for means for two genepools indicated that the average values for two genepools were significantly different. The *p*-values for Student’s t-test indicated that the difference in mean values of all the traits among two genepools was highly significant (Table 3).

**Table 3:** Average values of important traits and their *p*-values for significance of difference of means between two genepools

Trait	Mean value		Absolute difference of means	P-value (t-test)
	Indian genepool	European genepool		
Days to emergence (in days)	7.67	8.38	0.71	2.23E-05
Days to flowering (in days)	70.98	127.02	56.04	2.56E-33
Days to maturity (in days)	148.48	177.81	29.33	7.22E-25
Plant height (in cms)	177.31	197.17	19.86	5.26E-05
Siliqua length (in cms)	4.05	2.93	1.12	1.06E-13
Seeds per siliqua	12.67	11.90	0.77	0.00356
Thousand seed weight (in gms)	3.72	3.21	0.51	0.0133

### Diversity analysis and population structure using SNP markers

A total of 61 SNP markers widely distributed across the *B. juncea* genome were used for the characterization of core set to develop diversity profile of 80 genotypes. Out of 61 markers, 48 SNP markers were found to be polymorphic. Due to biallelic nature of the marker, a total of 98 alleles were amplified (Table 4). The minor allele frequency ranged from 0.00 to 0.46 with an average of 0.16. The gene diversity and heterozygosity also identified a remarkable degree of variability among the genotypes. The gene diversity value ranged from 0.013 to 0.49 and heterozygosity value ranged from 0.012 to 0.69 with an average of 0.16. PIC (Polymorphism Information Content) values in the present study were found to have ranged from 0.012 to 0.371 with an average of 0.19.

**Table 4:** Summary of SNP markers used for genetic diversity analysis

Marker	Minor Allele frequency	Gene diversity	Heterozygosity	PIC
A01_15062568	0.0733	0.1359	0.0667	0.1267
A01_1808370	0.3182	0.4339	0.0649	0.3398
A01_2139728	0.0385	0.0740	0.0769	0.0712
A01_6850903	0.2013	0.3216	0.4026	0.2699
A02_11859880	0.0130	0.0256	0.0000	0.0253
A02_24062658	0.3354	0.4458	0.6709	0.3465
A02_6601611	0.1795	0.2945	0.1538	0.2512
A03_20651981	0.3158	0.4321	0.1579	0.3388
A03_235511	0.0921	0.1672	0.1842	0.1533
A03_8547652	0.4423	0.4933	0.2436	0.3716
A04_17601178	0.3333	0.4444	0.0870	0.3457
A04_22058882	0.0068	0.0136	0.0137	0.0135
A05_77262	0.1597	0.2684	0.1528	0.2324
A06_13980299	0.0986	0.1777	0.0563	0.1619
A06_23478761	0.1800	0.2952	0.0933	0.2516
A06_6796237	0.3654	0.4638	0.0385	0.3562
A06_7120163	0.1159	0.2050	0.0580	0.1840
A07_11271	0.1776	0.2922	0.1184	0.2495
A07_15075686	0.2697	0.3940	0.3816	0.3164
A07_27294906	0.0455	0.0868	0.0390	0.0830
A08_19948782	0.0570	0.1074	0.0633	0.1017
A08_26316831	0.1859	0.3027	0.2436	0.2569
A08_3122114	0.0461	0.0879	0.0921	0.0840
A09_14703423	0.2961	0.4168	0.5921	0.3299
A09_21038191	0.3651	0.4636	0.3175	0.3561
A09_2675557	0.1169	0.2064	0.2338	0.1851
A09_53225827	0.0506	0.0961	0.0253	0.0915
A10_7119156	0.0316	0.0613	0.0127	0.0594
B01_31415063	0.2603	0.3851	0.1096	0.3109
B01_4700624	0.0132	0.0260	0.0263	0.0256
B02_14715231	0.1646	0.2750	0.1519	0.2372
B02_1692560	0.2500	0.3750	0.0897	0.3047
B02_372260	0.3882	0.4750	0.1974	0.3622
B03_11917496	0.0600	0.1128	0.0400	0.1064
B03_3641145	0.1842	0.3006	0.3684	0.2554
B03_36694310	0.0949	0.1718	0.0633	0.1571
B03_7368186	0.0063	0.0126	0.0127	0.0125
B04_1593069	0.0135	0.0267	0.0270	0.0263
B04_20687623	0.2273	0.3512	0.0909	0.2896
B04_27793042	0.2368	0.3615	0.3684	0.2962
B05_329343	0.1948	0.3137	0.2338	0.2645
B06_1587764	0.3467	0.4530	0.6933	0.3504
B06_18644772	0.2658	0.3903	0.5316	0.3141
B06_9741730	0.3333	0.4444	0.1600	0.3457
B07_19090096	0.4675	0.4979	0.9351	0.3739
B08_189749	0.1582	0.2664	0.1139	0.2309
B08_72248023	0.0584	0.1101	0.1169	0.1040
B08_7286923	0.3377	0.4557	0.0779	0.3631
Mean	0.1660	0.2410	0.1676	0.1978

**Fig. 2:**
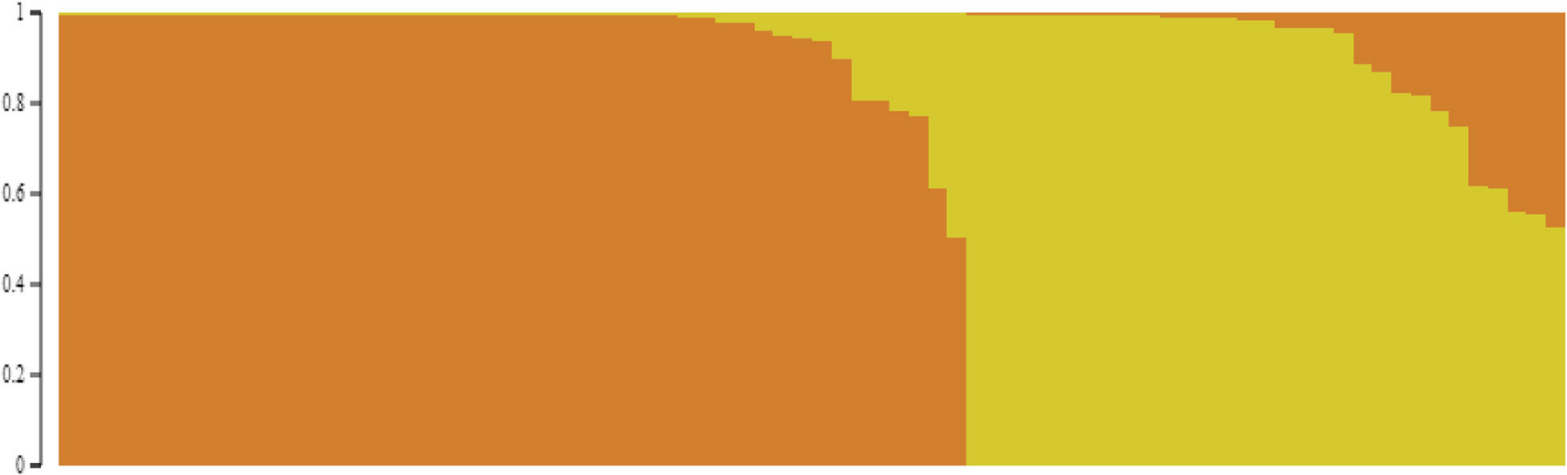
STRUCTURE analysis indicated genotypes grouping into two sub-populations based on membership coefficients indicated on vertical coordinate

**Fig. 3:**
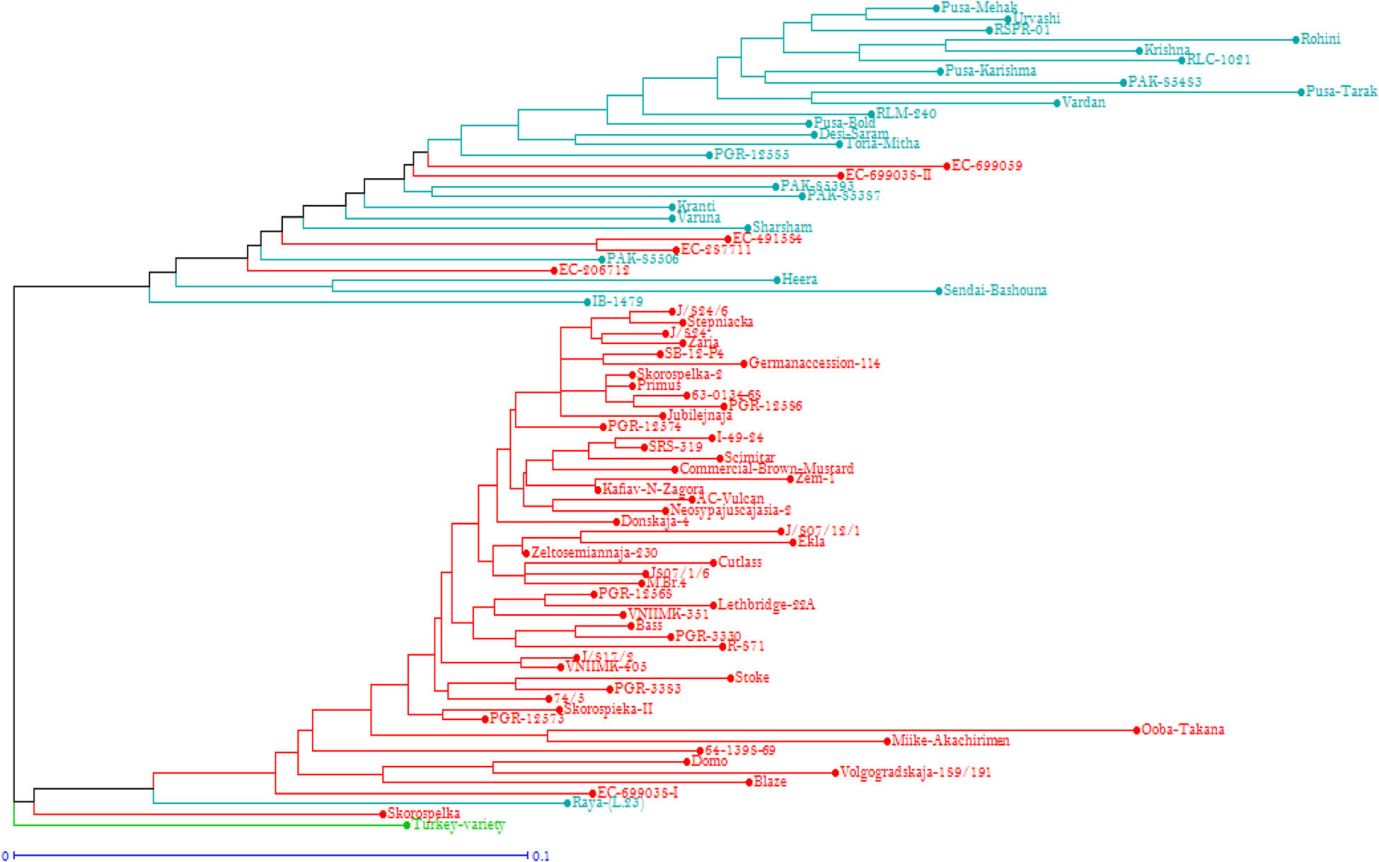
UPGMA dendrogram showing genetic relationship among 80 different *Brassica juncea* genotypes. Blue labeled genotypes are of Indian subcontinent, red labeled genotypes are of exotic origin and green labeled genotype is from Turkey

The population structure of 80 genotypes was estimated under the Hardy-Weinberg Equilibrium by using STRUCTURE V2.3.4 software. Based on the maximum likelihood and delta K (ΔK) values, the number of optimal groups was identified as two (Fig. 2). A dendrogram constructed using marker allelic data also grouped 80 genotypes into two distinct clusters and the local selection from Turkey forms a separate group. All 80 genotypes were grouped into three major clusters in which cluster I, II and III each contained 29, 50 and 1 genotypes, respectively. Cluster I and II also shows the grouping of genotypes into sub-clusters (Fig. 3). The clustering indicated the ability of SNP markers to group together the related genotypes from a geographical region with high level of accuracy. Cluster I consists of genotype mostly form Indian subcontinent and cluster II consists of exotic genotypes. However, some of the exotic genotypes (EC287711, EC206712, EC491584, EC699038-II and EC699059) were grouped along with the Indian genotypes which may be due to the fact that the allelic composition among these genotypes was identical at some of the loci that were considered in the present study. It may be possible to further refine their grouping patterns by characterization them at greater number of genetic loci as compared to the small subset of 61 SNP markers.

### Association mapping analysis

The association analysis to identify markers associated with six yield contributing traits was carried out using a set of 61 SNPs uniformly distributed across the all the chromosomes of *B. juncea*. In order to determine the true marker-trait associations, we used both *p* values and marker r^2^ value for association and only those significant associations are considered where the *p* values were < 10^− 6^. Out of 61 SNPs used, 18 SNPs were involved in 45 significant marker-trait associations for six different traits (Table 5). These associations were localized on 11 out of 18 chromosomes of *B. juncea* with a total of 23 marker-trait associations of A-genome and 22 of B-genome chromosomes. A highest of 16 associations were found for DTF, followed by 13 for SL, 12 for DTM, 2 for TSW and 1 each for SPS and PH. Almost all SNPs, except two – one each on A07 and B02, were involved in multiple associations with different traits. A lone SNP marker on B04 was found to be associated with four different traits; eight SNPs were found to be associated with three different traits followed by associations of six SNPs with two traits each. The SNP markers involved in associations with DTF, DTM and SL, were distributed on both A- and B-genome chromosomes, and these associations were found on multiple chromosomes. The SNPs for SPS, PH and TSW were found to be distributed on single chromosomes only of B-genome. The *p*-value for all the associations was less than the threshold value as determined by p-value (0.015) of false discovery rate. The *p*-values for all the associations ranged from 1.26E-05 to 1.15E-18 and the phenotypic variance contribution (r^2^) ranged from 0.20 to 0.89.

**Table 5:** Summary of marker-trait associations using SNP markers

S. No.	Trait	Marker	Chromosome	*p* value	r^2^
1.	DTF	A01_1808370	A01	7.59E-15	0.52
2.	DTM	A01_1808370	A01	2.69E-13	0.48
3.	SL	A01_1808370	A01	1.04E-10	0.41
4.	DTF	A01_6850903	A01	2.82E-08	0.30
5.	DTM	A01_6850903	A01	4.20E-07	0.26
6.	DTF	A02_24062658	A02	2.96E-06	0.22
7.	DTM	A02_24062658	A02	7.58E-06	0.20
8.	SL	A03_20651981	A03	1.53E-09	0.38
9.	DTM	A03_20651981	A03	4.59E-07	0.28
10.	DTF	A03_20651981	A03	7.37E-07	0.29
11.	DTF	A03_8547652	A03	4.16E-14	0.49
12.	DTM	A03_8547652	A03	1.05E-12	0.46
13.	DTF	A04_17601178	A04	2.27E-07	0.35
14.	SL	A04_17601178	A04	3.62E-06	0.27
15.	DTF	A06_23478761	A06	6.03E-06	0.26
16.	SL	A06_23478761	A06	7.11E-06	0.26
17.	DTM	A06_6796237	A06	8.28E-16	0.54
18.	DTF	A06_6796237	A06	1.94E-15	0.54
19.	SL	A06_6796237	A06	4.05E-12	0.45
20.	DTF	A07_11271	A07	4.34E-06	0.26
21.	SL	A08_26316831	A08	9.60E-08	0.31
22.	DTF	A08_26316831	A08	1.31E-07	0.31
23.	DTM	A08_26316831	A08	5.01E-07	0.28
24.	DTF	B01_31415063	B01	5.26E-09	0.36
25.	DTM	B01_31415063	B01	3.40E-07	0.30
26.	SL	B01_31415063	B01	5.96E-06	0.25
27.	SPS	B02_1692560	B02	126E-07	0.89
28.	DTF	B02_14715231	B02	2.10E-07	0.30
29.	SL	B02_14715231	B02	6.71E-06	0.24
30.	DTF	B04_20687623	B04	4.02E-12	0.45
31.	DTM	B04_20687623	B04	1.30E-09	0.37
32.	SL	B04_20687623	B04	1.74E-06	0.27
33.	PH	B04_20687623	B04	7.18E-06	0.26
34.	DTF	B06_18644772	B06	1.22E-14	0.49
35.	DTM	B06_18644772	B06	2.79E-11	0.39
36.	SL	B06_18644772	B06	6.61E-08	0.28
37.	DTM	B06_9741730	B06	1.15E-18	0.60
38.	DTF	B06_9741730	B06	2.22E-16	0.56
39.	SL	B06_9741730	B06	8.93E-11	0.42
40.	SL	B08_189749	B08	1.29E-07	0.31
41.	DTF	B08_189749	B08	8.26E-07	0.28
42.	TSW	B08_189749	B08	1.83E-06	0.30
43.	DTF	B08_7286923	B08	5.76E-17	0.56
44.	DTM	B08_7286923	B08	4.71E-15	0.51
45.	SL	B08_7286923	B08	8.20E-09	0.34

## Discussion

A plethora of molecular marker-based studies have led to a greater understanding of the genetic make-up of *Brassica* species*.* SNP markers have been vital for the (fine) mapping of genes of agronomic importance with the goal of implementing marker-assisted breeding of elite crop cultivars. SNPs are distributed far more frequently in a genome and have been used to develop high-density molecular genetic maps and fine mapping of a region of interest. The abundance of SNPs in genome, low mutation rate and high heritability offsets the disadvantage of bi-allelism. SNPs are found randomly distributed throughout the genome in both repetitive and non-repetitive regions, however those present in the genic/non-repetitive regions are of keen importance. The presence of orthologous regions among the progenitors of allopolyploid genome adds an extra layer of genome complexity in addition to repetitive elements. However, recent advances in reducing the genome complexity coupled with NGS technologies have been highly successful to develop genome-wide SNPs in crops.

In the current study, a pair of restriction enzyme digestion (*Mse*I and *Sac*I) was used for ddRAD sequencing of unique regions of *B. juncea*. The similar technique of genome complexity reduction has also been employed in several crops [22–24] animal [25] and insects [26, 27] species. A number of modifications of this technique have been proposed. In case of other polyploid crop (cotton), GR-RSC (Genome Reduction-Restriction Site Conservation) technique was followed and a combination of *Eco*RI and *Bfa*I restriction enzymes were used with a size selection between 450 and 600 bp [28] while another study preferred to use a combination of *Eco*RI and *Msp*I with size selection around 200–400 bp [25].

Following sequencing of genotypes, a total of 2300 MB paired-end sequence data were obtained from six *B. juncea* genotypes with an average of 383.33 MB from each genotype. Similarly an average of 147.3 MB data was obtained following dd-RAD sequencing of rice [29]. Considering the genome size of *B. juncea* of 955 MB and the single read sequencing data from six genotypes of 1150 MB, the individual genotype represent an average of 20% of the whole genome and thus, reducing the genome complexity by nearly five folds. Another study on Brassica species reported a reduction of nearly similar genome portion following ddRAD [30]. The mean quality score for both reads ranged from 34.63 to 35.40 and 90% sequence data with a Q score of at least 30 indicated that the sequencing reads were of high quality for reference genome alignment and SNP identification. Similar quality scores for high throughput sequencing runs have been reported with different genome complexity reduction method (SLAF-seq) in tea [31]. Due to high Q score, a large proportion (nearly 83%) of sequence reads were mapped to unique positions in the reference genome indicating the utility of ddRAD method to target unique regions in a genome. The mapping of reads to unique regions also ensured that the SNPs from duplicated or paralogous regions are excluded for further analysis.

Typically, the SNPs are distributed throughout a genome and the average frequency of distribution of SNPs has been found to be between 100 nt to 500 nt. In the present study, the occurrence of 93% of hypervariable regions (hotspots) of SNPs in the coding regions of *Brassica juncea* with SNPs distributed in upstream, downstream and in the intergenic regions of the coding regions. Most of these hypervariable regions had SNP frequency of less than 10 nt. Further, the detection of 40 genes/coding sequences in the chromosomal regions harbouring SNP hotspots might point to a possible regulatory role of these SNPs in the expression of these genes. Although, few previous studies have reported such SNP hotspots in repetitive regions mostly due to errors of DNA polymerase resulting in strand slippage and unequal exchange [32, 33] or due to presence of mutational hotspots or recombination hotspots [34]. The SNP hotspots along each chromosome were found to be distributed randomly and the number of SNPs involved in such hotspots ranged from four to eleven within 50 nt of chromosomal region in the current study. The role of high selection pressure due to environmental stress could lead to the accumulation of mutated allelic sites in the genic regions that improve survival of the crop under adverse environmental conditions [35, 36].

The high proportion (97%) of functional SNPs across a set of highly diverse genotypes indicated the accuracy of ddRAD technology to invariably target same locus across different individuals during the library preparation and partly due to the improved bioinformatics tools for sequence mapping and SNPs identification for complex and polyploidy crops. The SNPs identified through RAD-seq and its modifications in the previous studies have shown similar functionality levels in other crops as well [37, 38]. The biallelic data obtained from a subset of 61 functional SNPs in the present study was able to group diverse *B. juncea* genotypes into two major clusters- Indian and Exotic (European) genepool. The diversity and clustering results are in agreement with the previous studies based on SSR and other marker system. The SNP-based diversity analysis also concluded that a small subset of uniformly distributed SNPs would be highly useful for various genetic analyses.

The morphological characterization of six traits revealed very interesting patterns on correlation matrix. The bimodal distribution for DTF and DTM upon combined analysis of all the genotypes indicated that these two traits are controlled by different set of genes in Indian and European genepools. The European genepool has traditionally been domesticated under low-temperature short-day conditions while the Indian genepool is more conducive for sowing in moderate to low temperature conditions found mostly in the north-western plains of Indian subcontinent. The hypothesis of different set of genes controlling DTF and DTM in Indian and European genepools got further strengthened upon getting a unimodal distribution for DTF and DTM in correlation matrices individually for Indian and European genepools. However, the detailed interaction between the genotype and phenotype could be studied by undertaking QTL analysis and other genetic analyses.

In the present study, a common subset of 61 SNPs was used for diversity, population structure and association analyses. For diversity and population structure analyses, the subset of SNPs was able to group 80 genotypes into two distinct clusters, each over-represented by genotypes either from Indian and European (exotic) genepools; which indicated the usefulness of strategy involving the usage of sparse but uniformly localised SNPs for various genetic analyses.

A subset of SNPs representing all chromosomal regions of *B. juncea* was used to identify significant marker-trait associations. The association analysis using SNP subset was able to localize genes for various agro-morphological trait on different chromosomes, identifying genome regions for undertaking fine mapping of traits/genomic regions with large number of molecular markers. A majority of SNPs identified associations with multiple traits thus essentially indicating either the clustering of genes for multiple traits or involvement of same set of genes regulating multiple traits in the same genomic regions. Among these traits, DTF and DTM had invariably common SNP/genomic region associated with them, thus implying that the genes for these two traits are clustered together and/or likely have correlated/coordinated expression of genes. A recent study, using F_2_ mapping population, in *Brassica napus* has also identified the co-localisation of QTLs (and eQTLs) for flowering time and various growth-related morphological traits to a common genomic region of chromosome A10 [39]. In another study, QTLs for various quality and nutritional traits were again mapped to common regions of a genetic map of a DH (double haploids) mapping population in *Brassica napus* [40]. Such clusters of QTLs for multiple traits were also reported using chromosome segment substitution lines (cssls) in *Brassica rapa* [41]. High correlation between DTF and DTM traits, in the current study, among both Indian and European genepools also indicate the high probability of association of common genomic regions (and SNPs) for both the traits as reported in one of the earlier study as well [42].

The presence of a common ancestral genome between three polyploidy species led to the identification and comparison of association analysis results. In the current study, the associations for DTF were mapped to A- and B- genome chromosomes. Similarly genes for flowering time have been identified on both A- and B- subgenomes of *B. juncea* [43]. Two highly significant associations for DTF were identified each at 6.8 MB (A06_6796237) and 23.4 MB (A06_23478761) in the current study are in agreement with the results for flowering time related (FTR) genes. Thirty three flowering time related (FTR) genes were identified on chromosome A06 between 7.2 MB – 21.6 MB regions using transcriptome analysis [44]. The association analysis results of the current study indicated that a subset of sparse but uniformly localised SNPs would be highly useful to demarcate genomic regions for traits of interest.

## Conclusion

This is the first report of use of ddRAD-seq for the development of SNPs in *Brassica juncea*. The SNPs were developed initially from sequence comparison of six genotypes only; however the SNPs were found to be functional when tested on a diverse set of genotypes. The SNPs used for association analysis were also found to be significantly associated with six morphological traits. Given the fact that *Brassica juncea* has narrow genetic base, the SNPs identified in the current study would form an excellent source for various genetic studies including linkage mapping, fine mapping and association analysis.

## Methods

### Plant material and DNA extraction

A set of six *B. juncea* genotypes (three each from Indian and Exotic germplasm) were selected for use in ddRAD library preparation. *Pusa Tarak* (BJI-1), *Urvashi* (BJI-2) and *RSPR-01* (BJI-3) were selected from Indian genepool and *Zem 1* (BJE-1), *Donskaja IV* (BJE-2) and *EC287711* (BJE-3) were selected from European (exotic) genepool. Seeds were procured from (Dr. Deepak Pental) University of Delhi (South Campus), India and National Bureau of Plant Genetic Resources, New Delhi, India. SNP genotyping was performed on 80 diverse *B. juncea* genotypes that were procured from Plant Gene Resources, Agriculture and Agri-Food, Canada and Genetics & Plant Breeding Department, SKUAST-Jammu, India (Additional file 1:Table-S4). Total genomic DNA was isolated using modified SGS buffer method [45] and purified DNA was used for dd-RAD library preparation.

### Morphological data evaluation and statistical analysis

The phenotypic data of diverse core set of *B. juncea* was also recorded from two different locations in 2015–16 and 2016–17. The data were collected for six traits: days to flowering (DTF- number of days from sowing to the date when 50% of the plants had their flower opened in each plot), days to maturity (DTM- number of days from sowing to the date when pods on 75% of the plants in each plot were turned browned), plant height (PH- in meters), siliqua length (SL- in centimeters), seeds per siliqua (SPS- average number of the seeds present in single pod/siliqua) and thousand seed weight (TSW- weight in grams of the 1000 seeds collected in random). The traits value of each genotype was defined as an average of two replicates in the same location. The correlation coefficients between traits were determined using *Student’s t-test* and the variance components were also calculated.

### ddRAD library development and NGS sequencing

The ddRAD-seq protocol [25, 46] was used with slight modification for the construction of sequence-barcoded reduced representation libraries (RRLs) from six *Brassica juncea* genotypes. For ddRAD library preparation, ten microgram of purified DNA was digested to completion with *Mse I* and *Sac I*. The digested DNA was separated on 0.8% agarose gel; fragments between 300 and 400 bp were gel excised and eluted. The eluted and purified DNA was then end repaired, short dA-tail was attached and ligated with the adapters following manufacturer protocol. The ligated DNA was amplified using PCR to enrich and add the Illumina specific index and flow cell annealing sequences to the fragmented DNA. For each six genotypes, six different index sequences were used so at to facilitate the process of pooling. All six DNA samples were normalized to a final concentration of 50 ng/μl and pooled to reach a final volume of 300 μl to generate a reduced representation library. The pooled dd-RAD library was then sequenced using Illumina HiSeq 2000 to generate 100 bp paired-end reads.

### Sequence preprocessing and SNP detection

**Fig. 4:**
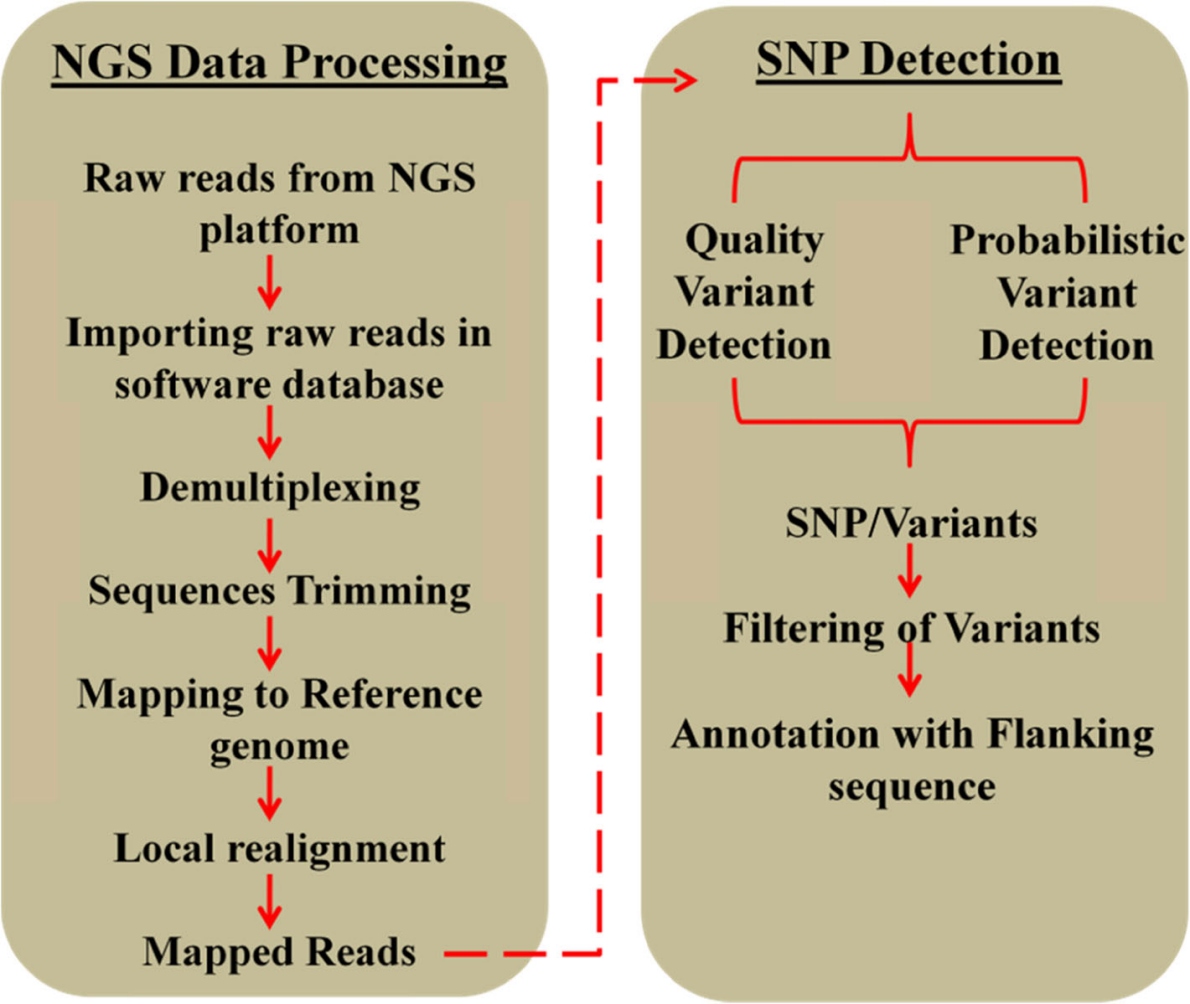
Workflow of various steps involved in SNPs identification

The ddRAD-seq reads obtained after sequencing were bioinformatically analyzed using CLC Genomics Software in order to obtain a high quality SNP set. The paired end sequencing reads were subjected to a series of steps (demultiplexing, trimming, mapping with reference genome, local realignment, SNPs detection and annotation with flanking sequences) through a pipeline. The following filtering scheme (Fig. 4) was used to maximize the retention of true genic polymorphic SNPs: (1) trimming of 13 bases from forward and 3 bases from reverse end, (2) mapping parameters were set to- mismatch cost: 2, insertion cost: 3, deletion cost: 3, length fraction: 0.5, similarity fraction: 0.95 and we have selected to perform local alignment instead of global alignment as it allows the ends to be left unaligned if there are many differences from the reference at the ends, (3) probabilistic SNP detection method was used for SNP detection from mapped reads with parameters- minimum coverage: 4, variant probability: 98.00 and ploidy: 2 and (4) flanking sequence of 400 bp. For mapping of reads, *Brassica juncea* genome was used as a reference genome [47].

### Validation of SNPs and genotyping

A subset of 61 SNP loci was selected with 3–4 SNPs from each chromosome and was validated across the diverse set of *B. juncea*. The sequences flanking each SNP were used to synthesize forward, reverse and *iPLEX* universal extension primer using Agena CXassay design suite V2.0 software. The forward and reverse PCR primers were diluted to the concentration of 100 μM, while *iPLEX* universal extension primers were diluted to the concentration of 500 μM. The experimental procedure included- (1) multiplex PCR using forward and reverse primer, (2) SAP (Shrimp Alkaline Phosphatase) clean up reaction, (3) *iPLEX* extended reaction with the amplified product, (4) resin cleanup reaction to remove salts, (5) spotting of primer extended product on spectro-chip and (6) spectro-chip detection using MALDI-TOF mass spectrometry. The genotype calls were evaluated through MassARRAY TYPER 4.0 software.

### Population and diversity analysis

The SNP genotyping data were used for population structure and genetic diversity analyses [48] following Singh et al. [49]. The posterior probabilities (*qK*) were estimated with 10,000 burn-ins followed by 100,000 iterations. For structure analysis, the diverse population was assumed to be following an admixture model and correlated allele frequencies with no prior population information. The structure analysis was performed with 5 replicates for each *K* ranging from 1 to 5. The *ΔK* was calculated using Structure Harvester software [50] to obtain an optimal value of *K.* The membership coefficient with a threshold of 70% for each replicate of structure analysis was used to generate a Q matrix using the software CLUMPP [51]; followed by plotting of Q matrix using DISTRUCT software [52]. The polymorphic information content (PIC) value and allele frequencies were calculated using Powermarker v3.51 [53]. The unweighted neighbor joining tree method was implemented in Darwin5 software [54] for constructing a phylogenetic tree; and the bootstrap value for this tree was determined by re-sampling loci at 1000 times.

### Gene identification and annotation using database

Flanking sequence of SNPs/ hyper-variable regions were compared against the *B. juncea* database using BLASTX (cutoff E-value of 1E-10) to identify the corresponding sequences in the protein database [55].

### Association analysis

Association analysis was performed by using the genotypic (SNPs) and phenotypic data of the diverse *Brassica* genotypes and population structure data (Q matrix) by using TASSEL software [56]. Marker–trait association analysis was conducted using TASSEL 3.0 software along with the GLM procedure keeping significant threshold for the association at *P* < 0.01.

## Supplementary information

**Additional file 1: Figure S1.** Workflow design for SNPs/Variants detection in CLC Genomics Workbench (Red solid lines indicate input file; blue dotted lines indicate output file). **Figure S2.** Bioanalyser analysis of the prepared library (Blue peaks indicate ladder peaks and red peaks at 35 and 10,380 bp indicate internal standards). **Table S3.** Summary of ddRAD-sequence data for six genotypes. **Table S4.** Details of *Brassica juncea* genotypes used for association analysis.

## Data Availability

SNPs identified from six genotypes in this study have been submitted to the European Variation Archive at EMBL-EBI database under accession number PRJEB26751.

## Abbreviations

BJE: *Brassica juncea* Exotic
BJI: *Brassica juncea* Indian
ddRAD: double digest-Restriction Associated DNA
DH: Double Haploids
DTF: Days to flowering
DTM: to maturity
GLM: Generalized Linear Model
LD: Linkage Disequilibrium
MALDI-TOF: Matrix-Assisted Laser Desorption/Ionization-Time Of Flight
MAS: Molecular Assisted Selection
PCA: Principal Component Analysis
PH: Plant Height
PIC: Polymorphism Information Content
QTL: Quantitative Trait Loci
RRLs: Reduced Representation Libraries
SL: Siliqua Length
SLAF-seq: Specific Locus Amplified Fragment Sequencing
SNPs: Single Nucleotide Polymorphisms
SPS: Seeds per Siliqua
SSRs: Simple Sequence Repeats
TSW: Thousand Seed Weight
