# Supplementary material for: ddRAD sequencing-based identification of inter-genepool SNPs and association analysis in Brassica juncea
Source: BMC Plant Biol. 2019 Dec 30;19:594. doi: 10.1186/s12870-019-2188-x (PMC6937933; doi:10.1186/s12870-019-2188-x)
Supplement: Supplementary file 1 — Additional file 1: Figure S1. Workflow design for SNPs/Variants detection in CLC Genomics Workbench (Red solid lines indicate input file; blue dotted lines indicate output file). Figure S2. Bioanalyser analysis of the prepared library (Blue peaks indicate ladder peaks and red peaks at 35 and 10,380 bp indicate internal standards). Table S3. Summary of ddRAD-sequence data for six genotypes. Table S4. Details of Brassica juncea genotypes used for association analysis. [file 12870_2019_2188_MOESM1_ESM.docx]

**Additional Files**

**
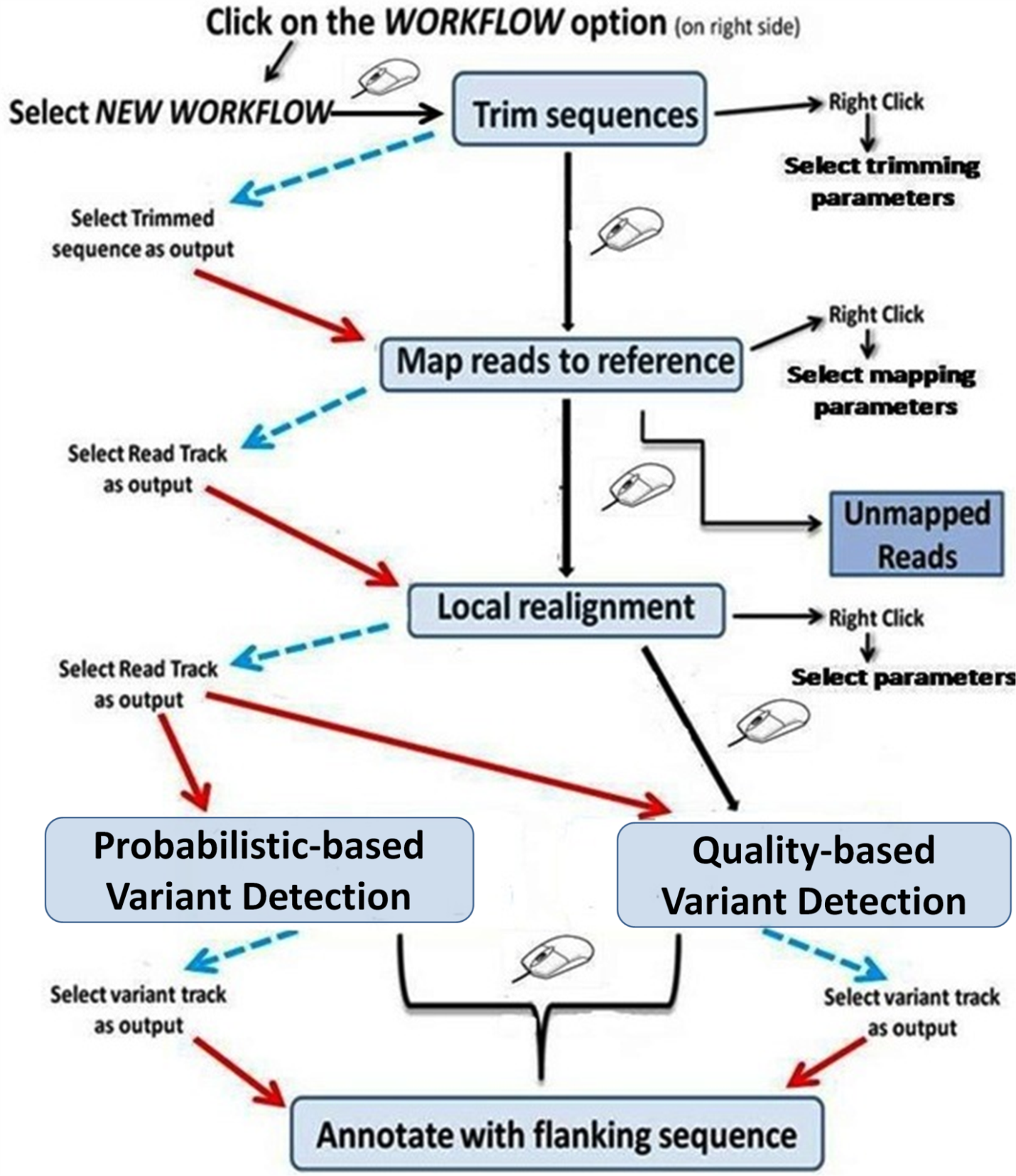
**

**Figure S1. Workflow design for SNPs/Variants detection in CLC Genomics Workbench**

**(Red solid lines indicate input file; blue dotted lines indicate output file;
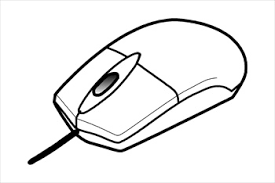
 -indicate right click on mouse)**


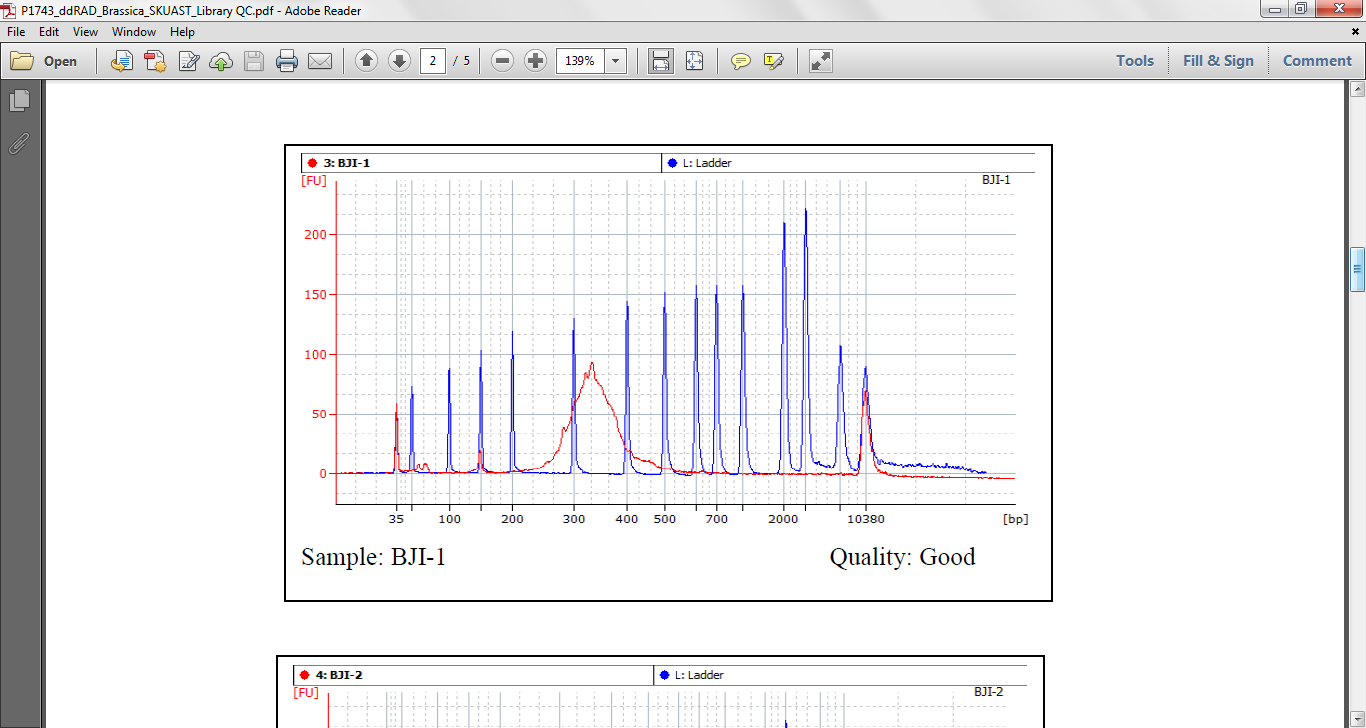

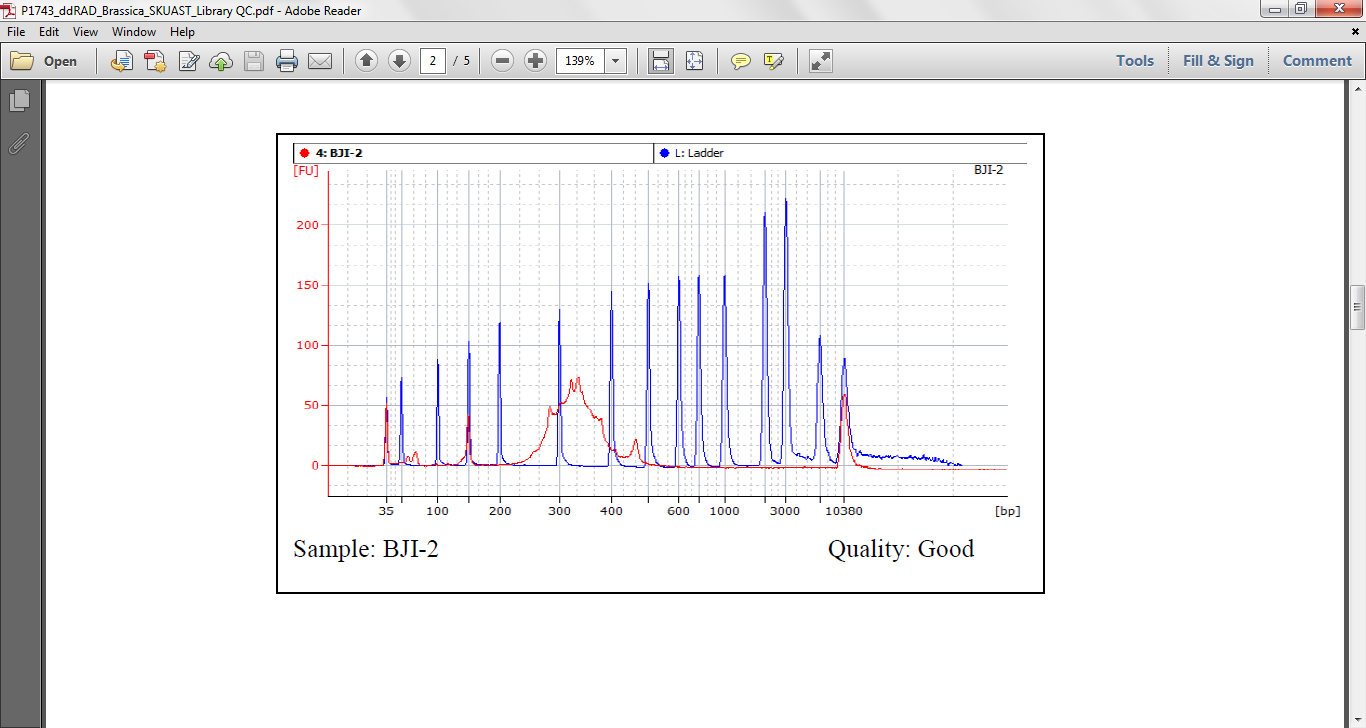


(a) (b)


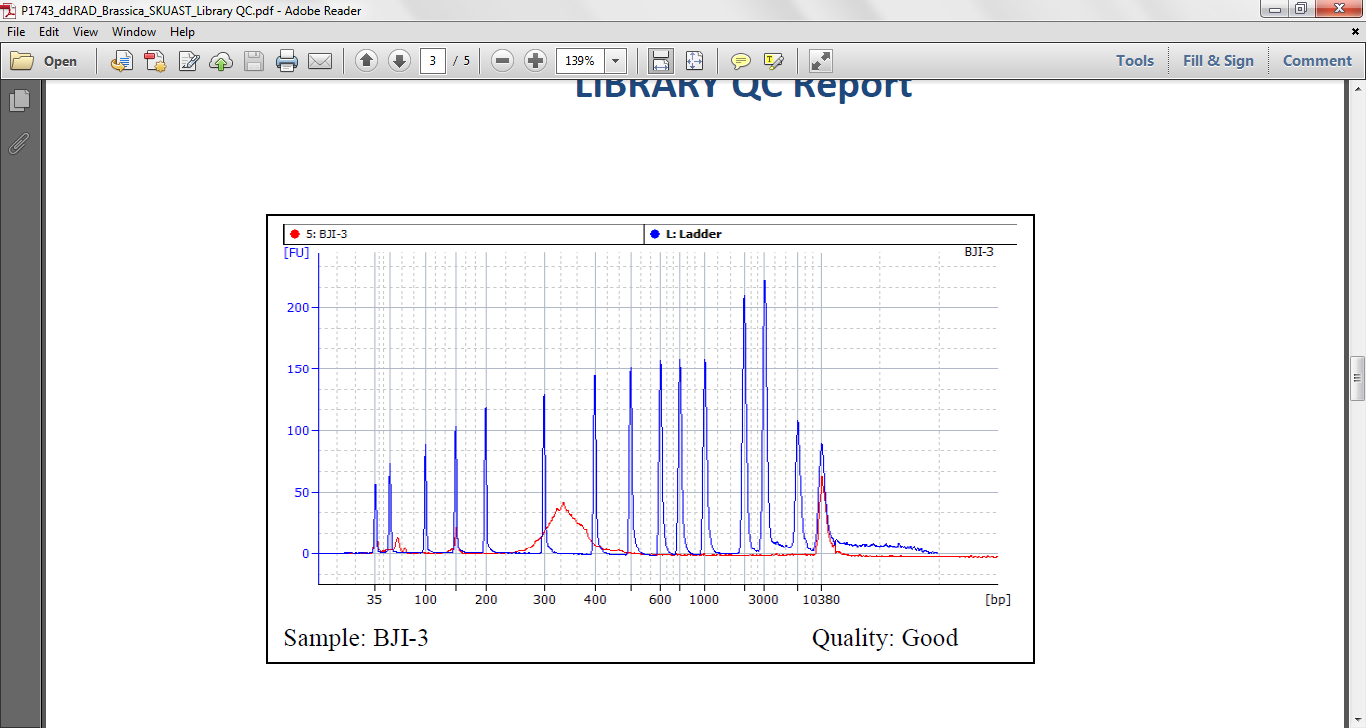

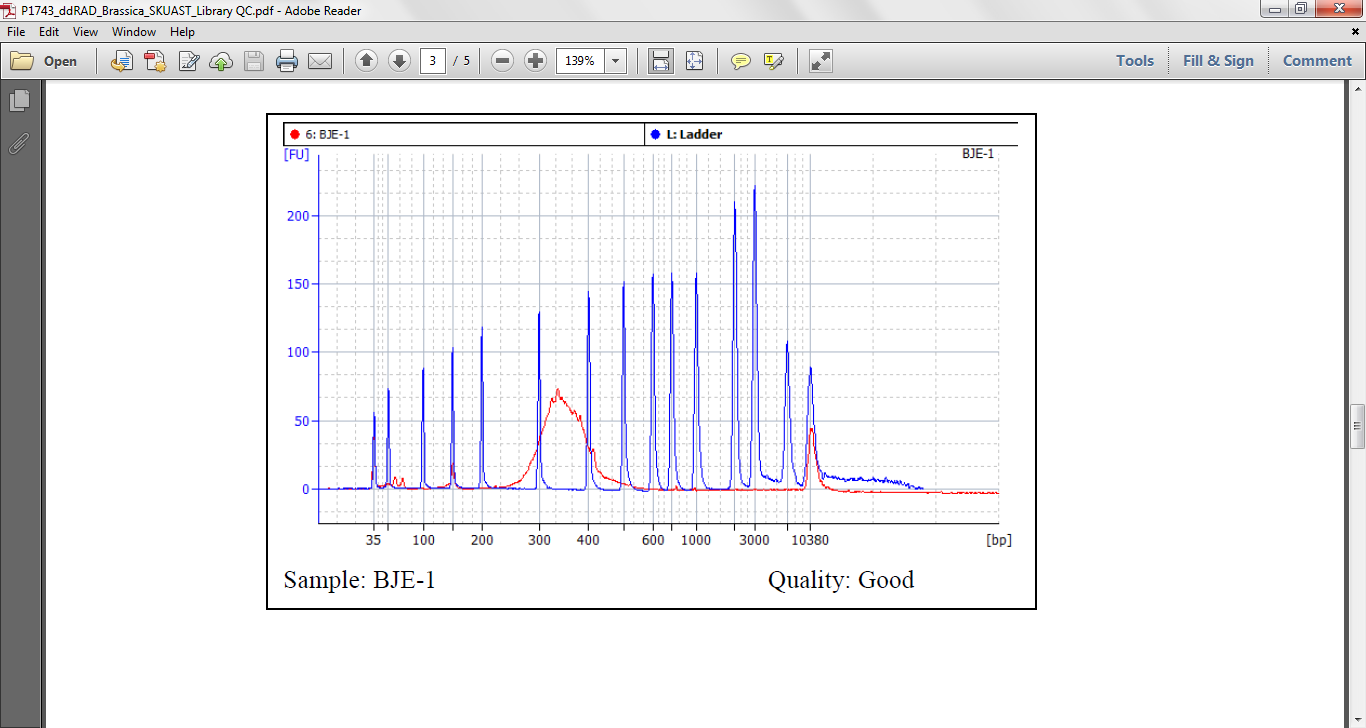


(c) (d)


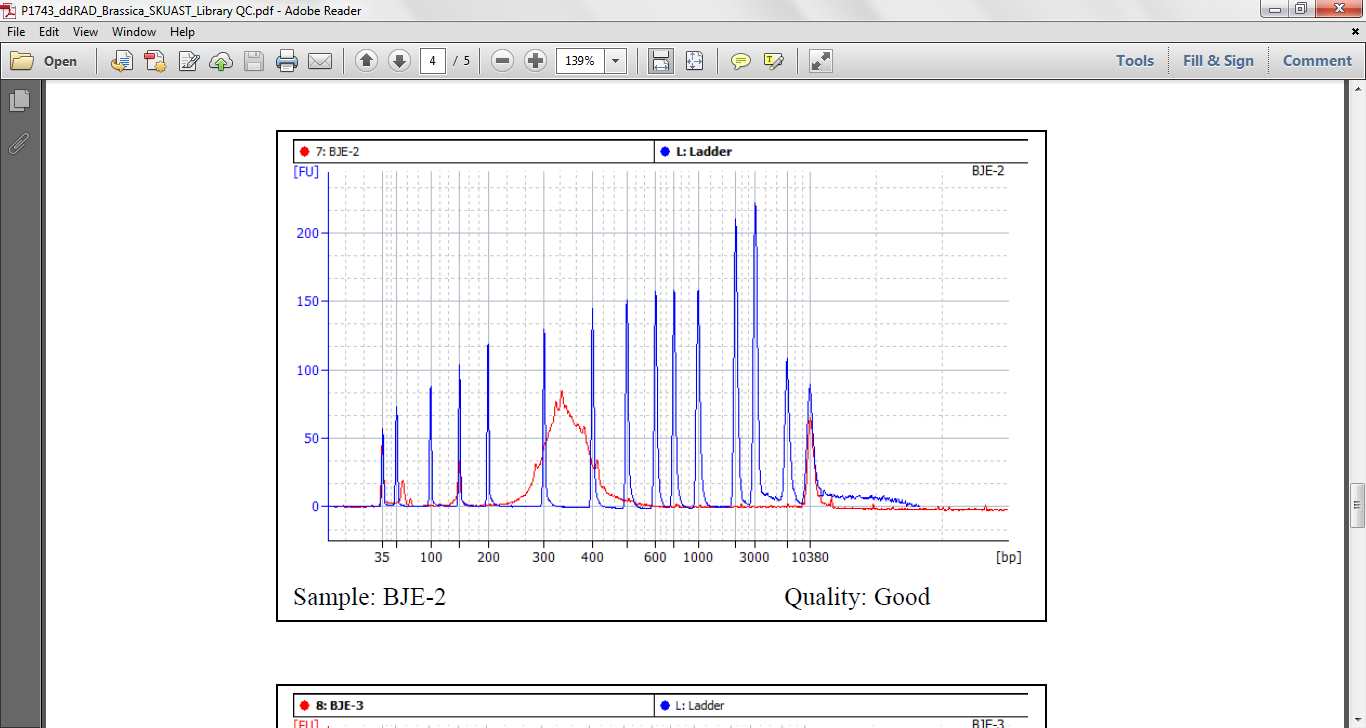

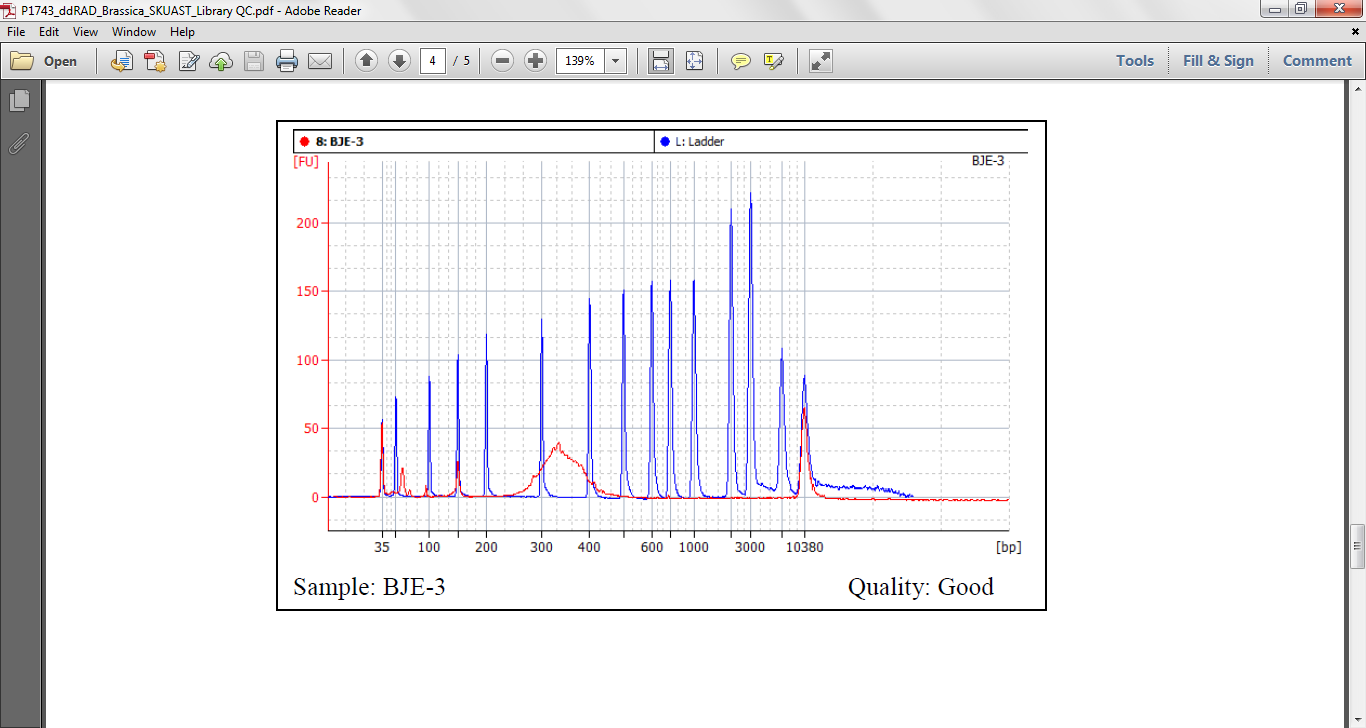


(e) (f)

**Figure S2. Bioanalyser analysis of prepared library for next-generation sequencing for six genotypes. Blue peaks indicate ladder peaks and red peaks at 35 and 10380bp indicate internal standards (BJI-1= Pusa Tarak, BJI-2= Urvashi, BJI-3= RSPR-01, BJE-1= Zem 1, BJE-2= Donskaja IV, BJE-3= EC 287711).**

**Table S3. Summary of ddRAD-sequence data for six genotypes**

| **SampleggSSAG**  **Genotypes** | **Read type** | **Mean read quality (Phred score)** | **Number of reads** | **%GC** | **%Q<10** | **%Q 10-20** | **%Q 20-30** | **%Q>30** | **Number of bases (MB)** | **Mean read length** |
| --- | --- | --- | --- | --- | --- | --- | --- | --- | --- | --- |
| ZemI (BJE-1) | R1 | 35.17 | 1155331 | 45.52 | 2.31 | 1.38 | 6.75 | 89.57 | 115.53 | 100.00 |
|  | R2 | 34.67 | 1155331 | 40.61 | 3.93 | 1.49 | 6.36 | 88.21 | 115.53 | 100.00 |
| Donskaja IV  (BJE-2) | R1 | 35.16 | 1180839 | 45.57 | 2.31 | 1.39 | 6.78 | 89.52 | 118.08 | 100.00 |
|  | R2 | 34.63 | 1180839 | 40.86 | 3.96 | 1.52 | 6.46 | 88.06 | 118.08 | 100.00 |
| EC-287711  (BJE-3) | R1 | 35.18 | 1731438 | 45.69 | 2.31 | 1.38 | 6.75 | 89.57 | 173.14 | 100.00 |
|  | R2 | 34.68 | 1731438 | 41.12 | 3.87 | 1.50 | 6.41 | 88.21 | 173.14 | 100.00 |
| Pusa Tarak  (BJI-1) | R1 | 35.26 | 3760684 | 45.53 | 2.23 | 1.35 | 6.56 | 89.86 | 376.07 | 100.00 |
|  | R2 | 34.73 | 3760684 | 41.21 | 3.83 | 1.48 | 6.27 | 88.42 | 376.07 | 100.00 |
| Urvashi  (BJI-2) | R1 | 35.41 | 1865540 | 43.11 | 2.01 | 1.28 | 6.30 | 90.42 | 186.5 | 100.00 |
|  | R2 | 34.92 | 1865540 | 41.07 | 3.47 | 1.42 | 6.04 | 89.07 | 186.5 | 100.00 |
| RSPR01  (BJI-3) | R1 | 35.20 | 1810898 | 45.52 | 2.30 | 1.37 | 6.66 | 89.66 | 181.09 | 100.00 |
|  | R2 | 34.69 | 1810898 | 40.91 | 3.91 | 1.48 | 6.32 | 88.29 | 181.09 | 100.00 |

**Table S4. Details of *Brassica juncea* genotypes used for association analysis**

| **S. No.** | **Genotype** | **Country** | **Seed Color** | **Seed Size** |
| --- | --- | --- | --- | --- |
| 1 | Neosypajuscajasia 2 | Russia | Yellow | Medium |
| 2 | Volgogradskaja 189/191 | Russia | Brown | Medium |
| 3 | Skorospelka | Russia | Brown | Medium |
| 4 | VNIIMK 351 | Russia | Yellow | Medium |
| 5 | VNIIMK 405 | Russia | Yellow | Medium |
| 6 | Zeltosemiannaja 230 | Russia | Yellow | Medium |
| 7 | Zaria | Russia | Yellow | Medium |
| 8 | Donskaja 4 | Russia | Yellow | Medium |
| 9 | PGR 3330 | Finland | Brown | Medium |
| 10 | PGR 3383 | Romania | Brown | Medium |
| 11 | Commercial Brown Mustard | Canada | Brown | Medium |
| 12 | Lethbridge 22A | Canada | Yellow | Medium |
| 13 | Blaze | Canada | Brown | Medium |
| 14 | Domo | Canada | Yellow | Medium |
| 15 | AC Vulcan | Canada | Yellow | Medium |
| 16 | Miike Akachirimen | Japan | Brown | Small |
| 17 | Sendai Bashouna | Japan | Brown | Medium |
| 18 | Ooba Takana | Japan | Brown | Medium |
| 19 | Ekla | Canada | Brown | Medium |
| 20 | Primus | Canada | Brown | Medium |
| 21 | Stoke | Canada | Yellow | Medium |
| 22 | Jubilejnaja | Russian | Yellow | Medium |
| 23 | PGR 12568 | Russian | Yellow | Medium |
| 24 | 74/5 | UK | Yellow | Medium |
| 25 | PGR 12573 | Russian | Yellow | Small |
| 26 | PGR 12574 | Canada | Yellow | Medium |
| 27 | Stepniacka | Russian | Yellow | Medium |
| 28 | J807/1/6 | UK | Yellow | Medium |
| 29 | J/807/12/1 | UK | Yellow | Medium |
| 30 | J/817/2 | UK | Yellow | Medium |
| 31 | J/824 | UK | yellow | Medium |
| 32 | J/824/6 | UK | Yellow | Small |
| 33 | PGR 12586 | China | Yellow | Medium |
| 34 | Cutlass | Canada | Yellow | Small |
| 35 | Scimitar | Canada | Brown | Medium |
| 36 | SRS 319 | Czechoslovakia | Brown | Medium |
| 37 | I-49-24 | Turkey | Brown | Medium |
| 38 | M.Br.4 | France | Brown | Medium |
| 39 | 63-0134-68 | Spain | Brown | Medium |
| 40 | 64-1398-69 | Spain | Brown | Medium |
| 41 | Bass | NA | Yellow | Medium |
| 42 | R 871 | Romania | Brown | Medium |
| 43 | Kafiav N Zagora | Bulgaria | Brown | Bold |
| 44 | German accession No. 114 | Germany | Yellow | Medium |
| 45 | Zem 1 | Australia | Yellow | Medium |
| 46 | Skorospieka II | Russia | Yellow | Medium |
| 47 | EC 287711 | Sweden | Brown | Medium |
| 48 | EC 491584 | Sweden | Brown | Medium |
| 49 | EC 206712 | France | Brown | Medium |
| 50 | EC 699059 | Spain | Brown | Medium |
| 51 | EC 699038-I | Spain | Brown | Medium |
| 52 | EC 699038-II | Spain | Brown | Medium |
| 53 | Local selection from Turkey | Turkey | Brown | Medium |
| 54 | PGR 12585 | Pakistan | Brown | Medium |
| 55 | PAK 85483 | Pakistan | Brown | Bold |
| 56 | PAK 85506 | Pakistan | Brown | Medium |
| 57 | PAK 85590 | Pakistan | Brown | Bold |
| 58 | PAK 85667 | Pakistan | Brown | Medium |
| 59 | Toria Mitha | Pakistan | Brown | Medium |
| 60 | Desi Saram | Pakistan | Brown | Medium |
| 61 | Sharsham | Pakistan | Brown | Medium |
| 62 | PAK 85393 | Pakistan | Brown | Medium |
| 63 | Raya (L.23) | Pakistan | Brown | Bold |
| 64 | SB-12-P4 | India | Yellow | Medium |
| 65 | RLM-240 | India | Brown | Medium |
| 66 | RLC 1021 | India | Brown | Bold |
| 67 | Krishna | India | Brown | Bold |
| 68 | Rohini | India | Brown | Bold |
| 69 | Vardan | India | Brown | Medium |
| 70 | IB 1479 | India | Brown | Medium |
| 71 | IB 1436 | India | Brown | Medium |
| 72 | Pusa Tarak | India | Brown | Bold |
| 73 | RSPR-01 | India | Brown | Bold |
| 74 | Urvashi | India | Brown | Bold |
| 75 | Pusa Bold | India | Brown | Bold |
| 76 | Pusa Mehak | India | Brown | Bold |
| 77 | Pusa Karishma | India | Brown | Bold |
| 78 | Varuna | India | Brown | Bold |
| 79 | Kranti | India | Brown | Bold |
| 80 | Heera | India | Yellow | Medium |
